# Laser-Machining of Microchannels in NiTi-Based Shape-Memory Alloys: Experimental Analysis and Process Optimization

**DOI:** 10.3390/ma13132945

**Published:** 2020-07-01

**Authors:** Muneer Khan Mohammed, Abdulrahman Al-Ahmari

**Affiliations:** 1 Raytheon Chair for Systems Engineering (RCSE), Advanced Manufacturing Institute, King Saud University, Riyadh 11421, Saudi Arabia; alahmari@ksu.edu.sa; 2 Industrial Engineering Department, College of Engineering, King Saud University, Riyadh 11421, Saudi Arabia

**Keywords:** NiTi, shape-memory alloys, laser, microchannel, optimization

## Abstract

Nickel–Titanium (NiTi)-based shape-memory alloys (SMA) are utilized in automotive, biomedical, microsystem applications because of their excellent shape memory effect, biocompatibility and super elastic properties. These alloys are considered difficult to cut—especially with conventional technologies because of the work hardening and residual stresses. Laser-machining is one of the most effective tools for processing of these alloys especially for microsystem applications. In this work, a thorough investigation of effect of process parameters on machining of microchannels in NiTi SMA is presented. In addition, a multi-objective optimization is carried out in order to find the optimal input parameter settings for the desired output performances. The results show that the quality of microchannels is significantly affected by input parameters. Layer thickness was found to have a significant effect on taper angle of the microchannel. Scan speed, layer thickness and scan strategy were found to have significant effects on both spatter thickness and top-width error, but in opposite directions. The multi-objective optimization-minimizing taper angle and spatter thickness revealed an optimal solution that was characterized by high frequency, moderate speed and low layer-thickness and track displacement.

## 1. Introduction

Shape-memory alloys have the distinctive ability to withstand large recoverable strains and regain their original shape after deformation either instantaneously or upon heating [1]. Among various SMAs, NiTi alloys are most popular because of their better workability and commercial viability. These alloys find applications in biomedical implants [2], MEMS (Micro-Electro-Mechanical-Systems), sensors, actuators and antennae [3] because of their high specific strength, toughness, biocompatibility, superior shape memory effect (SME), high wear and corrosion resistance properties [4]. These alloys have poor thermal conductivity and low effective elastic modulus which makes its machinability challenging especially with conventional technologies due to work hardening and residual stresses. Conventional machining of SMAs is difficult and often associated with high cutting forces, poor surface integrity, greater tool wear, variations in shape and properties of the material due to heat [5]. Research has been done investigating the influence of cutting tool material on the quality of machining, the means of improving the material removal rate (MRR) during turning and drilling of NiTi alloys [6]. Kaynak et al. [7] studied the effects of cryogenic cooling on tool-wear rate during machining of NiTi SMA. Cryogenic cooling was found to be an effective tool in reducing the tool-wear significantly at higher speeds in addition to reducing progressive flank wear at the nose region and notch wear at lower cutting speeds [7]. However, there are still many issues such as severe burr formation, cratering and phase structure transformation which make conventional machining less favorable for NiTi SMAs.

Nonconventional technologies like laser-beam machining (LBM) [8], electric-discharge machining (EDM) [9], water-jet machining [10] were found to be effective than conventional technologies for the processing of NiTi alloys. The medical devices such vascular stents are mostly fabricated using laser micromachining process [11]. EDM finds applications in machining of NiTi alloys for biomedical implants [12]. Water-jet machining is also a favorable route for machining of NiTi alloys owing to reduced mechanical and thermal damages to the material, but however it is not found to be effective especially for micro applications [10]. Lasers because of their multiple industrial applications in cutting, machining, polishing, and joining of materials have found considerable research interest for the processing NiTi SMAs than other technologies. Moreover, lasers are an invaluable tool in additive manufacturing that have also led to additive manufacturing of NiTi SMAs [13]. Laser wielding is the most common method for joining NiTi SMAs due to the lower spot size and reduced thermal effects than other joining processes [14]. Laser scribing and laser-shock peening have proven to be effective tools for micro-patterning of NiTi surfaces with potential applications in aerospace and biomedical [15]. Laser- induced micro indents were found to produce higher ratios of shape memory effect due to lower subsurface damage in contrast to other processes [16]. Studies have been done showing the effect of laser-process parameters on the functional properties of NiTi SMAs. In general, controlled-process parameters with lower heat input were found to be effective in preserving the functional properties of the NiTi SMAs [17].

Microchannels in NiTi alloys have various applications in microactuators, sensors [18], biomedical implants [19] and microfluidics. The trend towards miniaturization and NiTi applications in MEMS and biomedical devices, which demand accurate shape and size make laser micromachining an effective tool for machining of NiTi SMAs. Laser processing of materials offers various advantages, but however it is a complex process involving large number of factors such as laser power, laser spot size, pulse width, pulse frequency, pulse overlap, scan speed, layer thickness, scan strategy and assist gas pressure. The quality of machining is mostly affected by selection these factors. Therefore, the analysis of process parameters and process optimization is essential to have desired output performances characteristics. Studies have been done to systematically investigate the laser processing of materials in order to analyze the effect of various process parameters on the quality characteristics of the micro-features. Farasati et al. [20] studied the laser drilling of micro holes in Ti6Al4V alloy using response surface methodology. Laser power and pulse interval time were found to influence the dimensional accuracy and MRR. Process optimization was carried out using desirability function approach to maximize MRR and minimize the taper of the micro holes. Optimal solution was characterized by high gas pressure, intermediate pulse power and frequency, maximum interval time and low pulse width. In multi scan laser-machining, the scan strategy also influences the performance characteristics. Leone et al. [21] investigated the effect of scan strategy, laser power and scan speed on the laser milling of alumina ceramic. Net scan strategy and low scan speeds were found favorable for better surface integrity. Multi-objective optimization maximizing MRR and minimizing surface roughness during laser milling of aluminum alloy was characterized by intermediate values of scan speeds and frequency [22]. Studies related to microchannels fabrication using different types of lasers on a variety of materials revealed the dependence of quality and geometry of microchannel on the suitable selection and combination of process parameters [23]. laser with shorter wavelengths were found to give precise control of the microfeatures and higher ablation efficiency due to better absorptivity of the material especially in case of transparent and hard materials [24]. Laser power and pulse repetition frequency were found to have a significant effect on the microchannel dimensions. Karazi et al. [25] incorporated artificial neural network and design of experiment methodology in order to predict the microchannel dimensions with respect to laser power and frequency during laser-machining of glass. Benton et al. [26] studied the fabrication of microchannels in PMMA using finite element modeling and experimental analysis. laser power and scan speed were found to have significant effect on the depth and width of the microchannel. In general, laser-process parameters were found to have opposite effect on dimensional accuracy and surface integrity. high pulse frequencies and high speed were found to be optimal for depth and width accuracy. Whereas, low pulse frequency and high pulse intensity were found to be favorable for surface integrity [27]. Therefore, it is evident that work has been done with regard to the investigations of laser-process parameters on the quality of microchannels in variety of materials. However, limited work has been done in the field of multi objective optimization especially considering laser scan strategy and microchannel quality characteristics such as taper and spatter.

In this work the effect of laser-process parameters on the quality of microchannels is investigated systematically using experimental design methodology. After the analysis and interpretation of the results, a multi objective optimization is carried out in order to find the optimal parameter setting which results in microchannels with minimum taper and spatter.

## 2. Material and Methods

**Figure 1:**
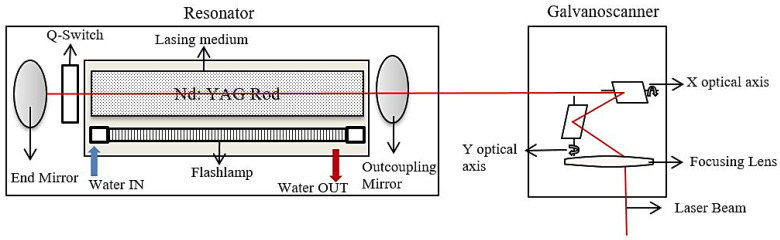
Schematic diagram of the laser setup.

The setup used in this work is the Lasertech 40 from DMG/Sauer (Geretsried, Germany) equipped with the Q-switched Nd:YAG laser operating with a wavelength of 1064 nm. The aperture size and laser pulse width were kept constant at 30 µm and 10 µs, respectively. The beam was operated in fundamental Gaussian mode (TEM_00_) with a maximum average power of 10 W. Figure 1 shows the schematic diagram of the setup. The laser beam is fed towards the sample and maneuvered in XY directions with the help of scanning system consisting of lens and glavanoscanner. The NiTi (Ni 50% Ti 50%) sheet of dimension 40 mm × 40 mm × 1 mm was used as the workpiece material.

**Figure 2:**
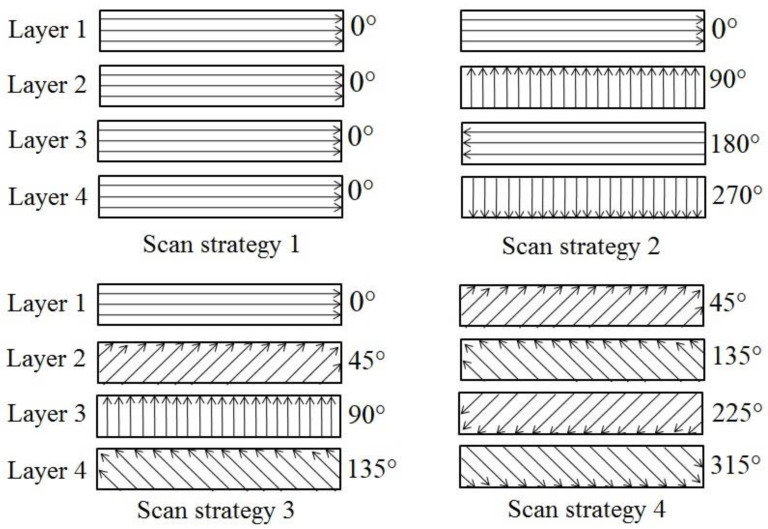
Scan strategies in terms of laser hatch line angles.

**Figure 3:**
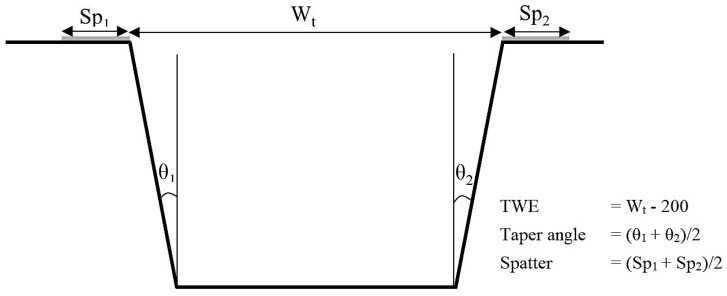
Schematic diagram of the microchannel cross-section detailing the output responses.

Design of experiment methodology was used for the parametric study and optimization of laser-process parameters for efficient and accurate machining of microchannels. After the initial screening experiments, five factors and their respective levels were selected as shown in Table 1. Frequency, scan speed, layer thickness and track displacement were numeric and have three levels each whereas scan strategy was the text factor with four levels. Scan strategy used in this work is in terms of hatch angles of the laser scans as shown in Figure 2 based on a previous research [28]. The laser scans the surface based on the set hatch angles for four consecutive layers, and these steps are repeated till the complete scan. In order to achieve the desired layer-thickness at a particular combination of speed, frequency and track displacement, preliminary tests were carried out to set the laser intensity. For each experimental run, the actual layer-thickness removed was measured by using the inbuilt touch probe. This was repeated until the error between the desired and actual layer-thickness in three successive layers was within the acceptable range. The output responses considered were top-width error (TWE), taper, spatter and MRR. Figure 3 shows the schematic diagram of the microchannel cross-section along the details regarding the output responses. TWE is the difference between the measured width at the top of the channel and the actual designed width (200 µm). Taper angle is the average of angles made by the side walls of the microchannel with the vertical. Spatter is the average of spatter thickness on both sides of the microchannel. A total of 65 runs were obtained for the selected factors and their levels using Optimal Design IV. The machined channels were cut through the cross-section using isomet-1000 precision cutter (Buehler, Lake Bluff, IL, USA) and mounted in the epoxy molds such that cross-sections face upwards. The samples were then grinded, polished and later viewed under the scanning electron microscope (SEM) (JEOL JSM-6610LV, Tokyo, Japan) in order to analyze the cross-section of the microchannel. The microhardness measurement were carried out on a Vickers microhardness tester (Struers DuraScan, Ballerup, Denmark) using a load of 200 g and a dwell time of 15 s.

## 3. Results and Discussion

**Figure 4:**
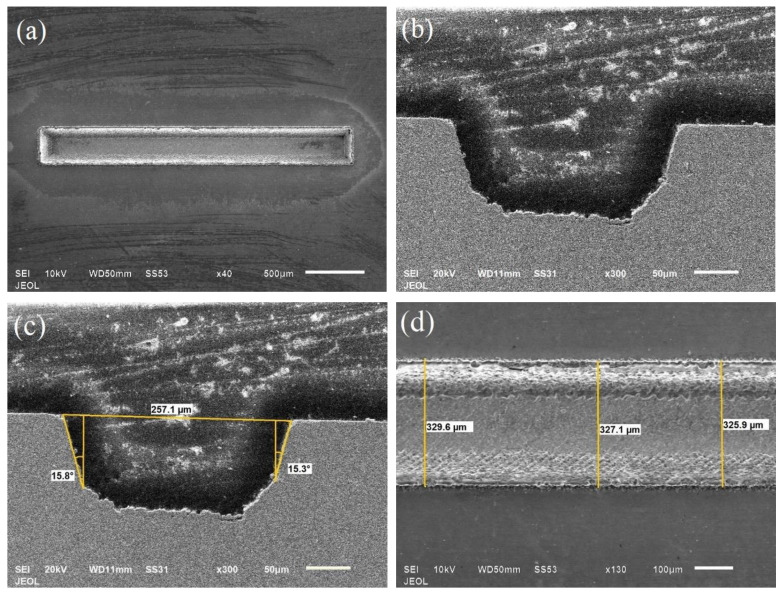
SEM images of the microchannel (**a**) Top view of the complete microchannel (**b**) cross-sectional image (**c**) measurement of TWE and taper (**d**) spatter measurement.

Rectangular cross-section channels of 200-μm width and 100-µm depth were machined in SMA samples. Optimization of rectangular cross-section channels was done so as to find out the effect of laser-process parameters on the cross-sectional accuracy, spatter and MRR. Based upon the optimal design IV a total of 65 channels were machined with different parametric settings. The channels were found to be uniform throughout the length of the channel as shown in Figure 4a. Figure 4b shows the rectangular cross-section of microchannel. The measurement of top width, taper angle and spatter around the periphery of the channel is done as shown in Figure 4c,d, respectively. After the measurement of responses, the results were analyzed as shown in Table 2.

### 3.1. Top-Width Error (TWE)

Dimensional accuracy of the microchannel was evaluated in terms of channel top-width error (TWE). It is the difference between the actual measured width at the top of the channel and the designed width. Scan speed, layer thickness and scan strategy were found to have a significant effect on TWE. Whereas frequency and track displacement were found to have little or no effect on the TWE.

**Figure 5:**
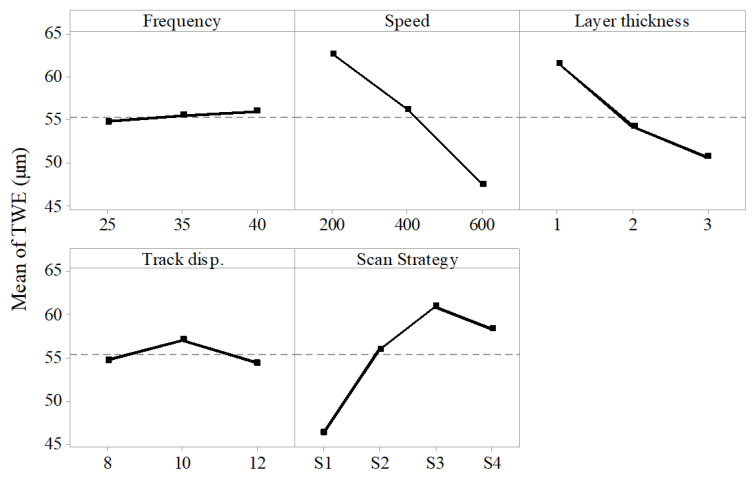
Effect of process parameters on top-width error (TWE).

Figure 5 shows the effect of process parameters on the TWE. In general, slower laser speed produces wider microchannel and hence higher TWE [29]. It is due to the fact that at low speeds the laser material interaction is longer, and the heat input is higher which results in overcutting [30]. As the layer thickness increases the dimensional error decreases, higher layer-thickness implies fewer total laser scans which favors the accuracy. Microchannel width is found to increase-with-increase in number of laser scans which in turn increases the TWE [31]. Scan strategy S1 which has the scan line parallel to the lamination direction was found to produce the microchannel with least dimensional error. This is due to the fact that Scan strategy S1 uses unidirectional scan pattern where as other strategies use the serpentine scan (snake scan) pattern which has more interaction with the edges and therefore results in overcutting.

### 3.2. Taper Angle

The taper angle of the microchannel is the average of the angles made by the vertical side walls of the microchannel with the perpendicular. The results show only layer thickness to be a significant factor effecting the taper angle. The frequency and scan strategy were found to have a little effect on the taper. Whereas speed did not have any effect on the taper.

**Figure 6:**
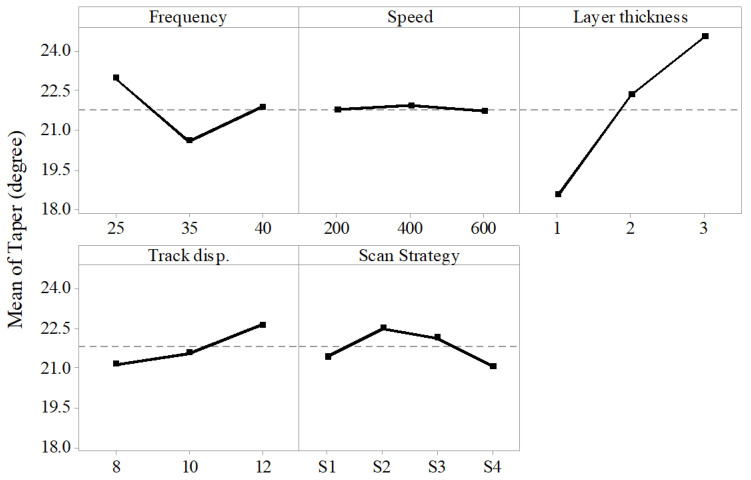
Effect of process parameters on taper angle of the microchannel.

**Figure 7:**
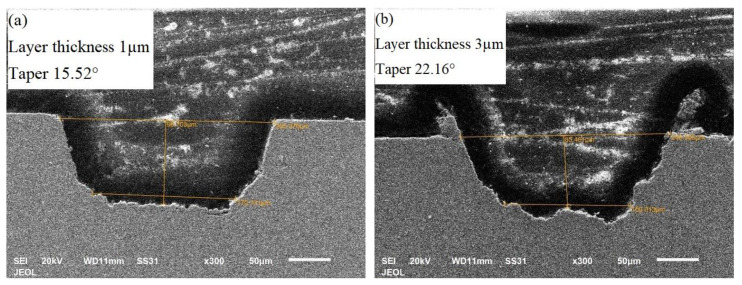
SEM images of the microchannel cross-section showing the variation of taper angle with layer thicknesses of (**a**) 1 µm and (**b**) 3 µm.

Figure 6 shows the effect of laser-process parameters on the taper angle of the microchannel. It could be seen that the taper angle increased almost linearly with the layer thickness and it was in agreement with the previous work [32]. The other factors had a little or no effect on the taper. Figure 7 presents the cross-sections of the microchannels fabricated by varying layer thickness and keeping other parameters constant. It can be seen that the layer thickness of 1 µm resulted in microchannels with less taper than the one with higher layer thickness. This was because the layer thickness had a direct relationship with the laser intensity. It was observed that taper angle increased with an increase in laser intensity [33]. As the layer thickness increased, the unit material required to be removed per laser scan increased, which was accomplished with increased laser intensity. The resultant higher molten metal eroded the side walls of the microchannels as it gets ejected out of the channel, resulting in tapered side walls of the microchannel [34]. Moreover, once some of the molten metal get adhere to the side walls, the laser loses the focus near the edges for the subsequent layers resulting in a tapered microchannel. Scan strategy S4 and scan strategy S1 were found to produce microchannels with less taper than the other two strategies. This was because of the presence of 90° hatch lines in both S2 and S3 strategies, wherein hatching was performed across the cross-section which did not ease the efficient ejection of molten material.

### 3.3. Spatter

Spatter is the ejected molten material that adhere to the periphery of the microchannel. It was measured using the top view of the microchannel at various places and the average was analyzed. The results show that scan speed, layer thickness and scan strategy had a significant effect on spatter. Frequency and track displacement were found to have a little or no effect on spatter.

**Figure 8:**
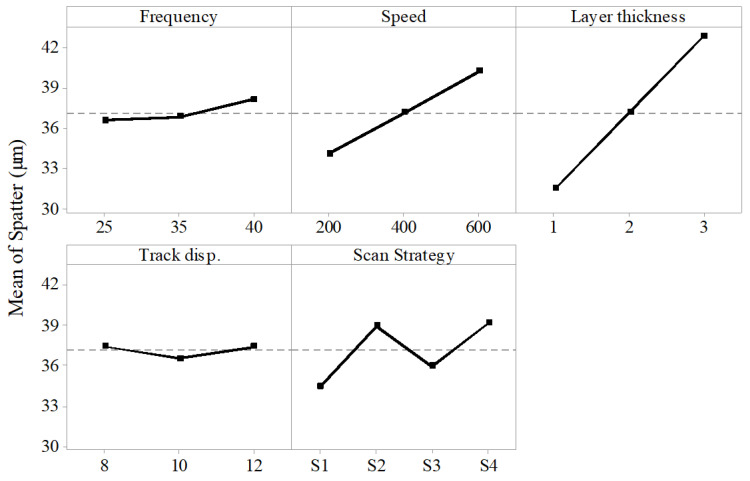
Effect of process parameters on the spatter thickness.

**Figure 9:**
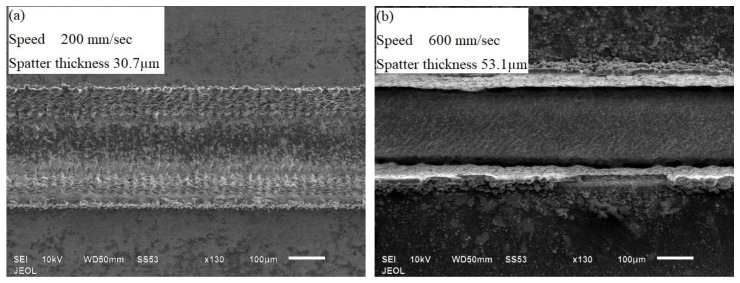
SEM images of the microchannel showing the variation of spatter with scanning speeds of (**a**) 200 mm/s and (**b**) 600 mm/s.

The effect of process parameters on the spatter thickness is shown in Figure 8. It could be seen that as the scan speed and layer thickness increased, spatter thickness increased. The increase was sharp in case of layer thickness than scan speed. Scan strategy S1 which had the scan line parallel to the lamination direction was found to produce the channel with least spatter thickness. As the layer thickness increased, the input energy and the unit material removed per laser scan increased. The laser therefore melted higher volume of material per scan which adhered to the top layer of the microchannel during ejection—resulting in increased spatter. Figure 9 shows the top view of the microchannels fabricated with different speed while keeping other parameters constant. It can be seen that the microchannel fabricated with low speed of 200 mm/s resulted in less spatter thickness than the one fabricated with high speed of 600 mm/s. This was because at higher speed the material removal rate and the melt pool pressure were higher due to which the molten material dispersed rapidly and became adhered to the periphery of the microchannel.

### 3.4. MRR

**Figure 10:**
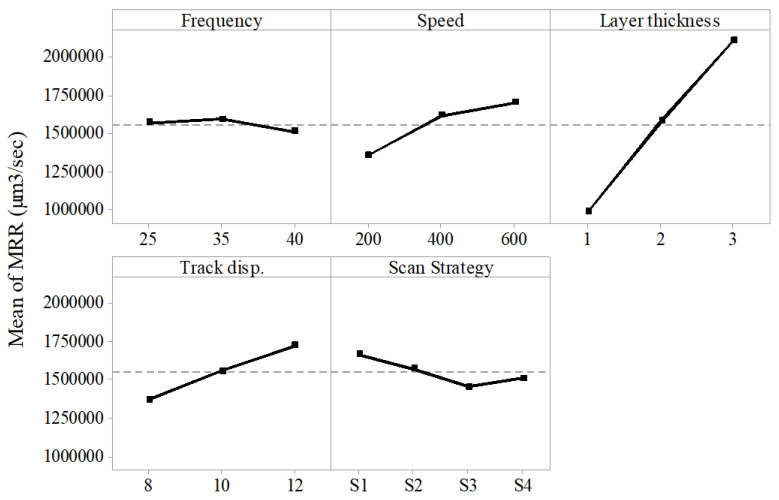
Effect of process parameters on MRR.

Material removal rate is the actual volume of material removed over machining time. The machining time is the sum of individual layer scan time in second, which is influenced by scan speed and track displacement. The analysis of results showed scan speed, layer thickness, track displacement and scan strategy had a significant effect on the material removal rate. In contrast, frequency was had a little or no effect on MRR. Scan speed, track displacement and layer thickness had a similar trend with the MRR. As these factors increased the MRR also increased; the increase was however significantly higher in case of layer thickness as shown in Figure 10. This was because as the layer thickness increased the depth of material removed per unit scan increased. Moreover, with higher layer thickness the total number of laser scans reduces drastically, which resulted in reduced machining time and hence in an increase in MRR. Higher scan speed and track displacement resulted in reduction of individual layer scan time and hence decrease in total machining time, which in turn increased the MRR. Scan strategy S1 had higher MRR than other strategies; this was due to the reduced scanning time in case of scan strategy S1, and is in agreement with the previous work [22].

### 3.5. Microhardness Analysis

**Figure 11:**
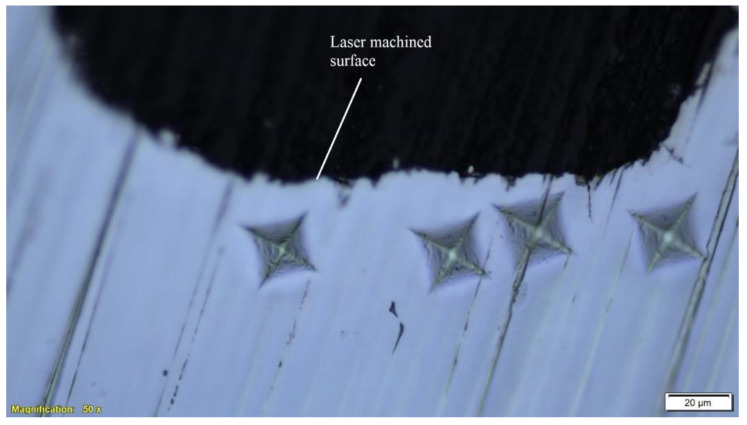
Microhardness indentations beneath the laser machined microchannel.

**Figure 12:**
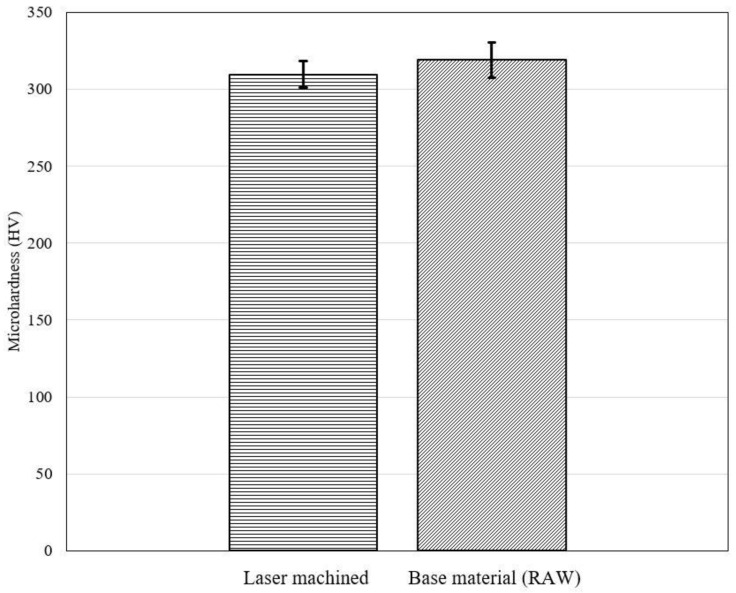
Microhardness comparison between laser machined surface and base material.

In order find the effect of laser processing on the mechanical properties of the material, microhardness tests were carried out beneath the microchannel surface as shown in Figure 11. The microhardness results of the laser machined surface were compared with that of base material. The results were found to be comparable as shown in Figure 12. The microhardness of the base material was found to be in the interval 319 ± 8 HV, whereas near the laser machined surface was found in the interval 310 ± 11. These results suggest the absence of any detrimental effect of the laser processing on the microhardness of the NiTi SMAs.

### 3.6. Optimization

**Figure 13:**
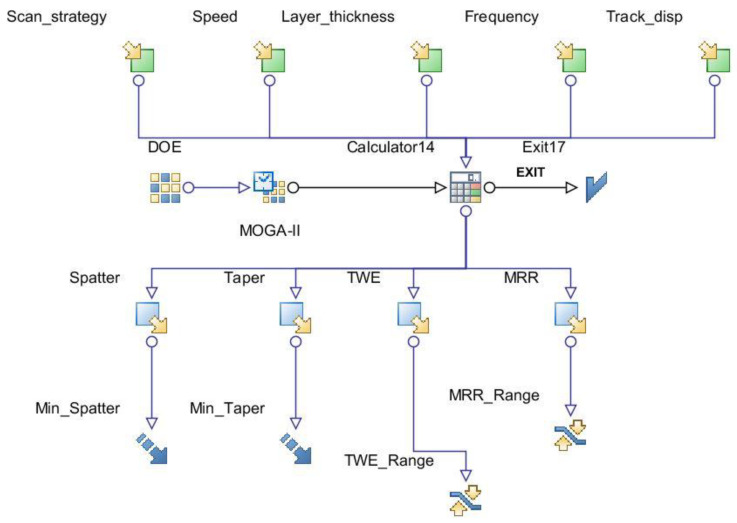
Optimization model workflow in ModeFRONTIER.

Multi objective optimization was performed in order to find the optimal parameters minimizing taper angle and spatter around the periphery of the microchannel while keeping the TWE and MRR as constraints within a range as shown in Table 3. Multi objective optimization is carried out using multi objective genetic algorithm (MOGA-II) and RSM in modeFRONTIER^®^ software (Version 4.0, Esteco SPA, Trieste, Italy), the optimization workflow is shown in Figure 13.

**Figure 14:**
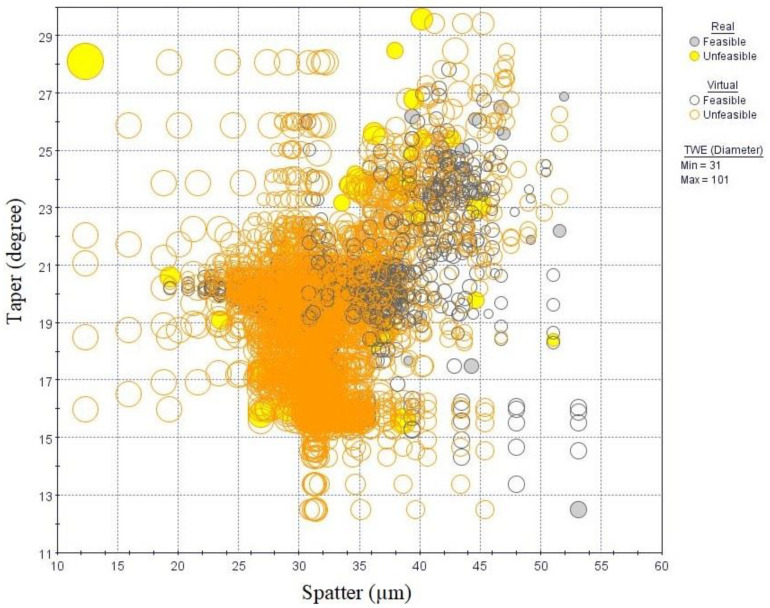
Three dimensional bubble chart with complete solution space.

MOGA-II is an effective algorithm that uses smart elitism for multi-objective search. The elitism operator prevents convergence to local optima. The algorithm considers a total of number of evaluations which are DOE runs multiplied by number of generations. In the current work, DOE runs were 65 and number of generations were set to 1000 in MOGA-II. Therefore, about 65,000 evaluations were carried out during optimization. Of these, about 38% of the results were found to be unfeasible due to violation of constraints. The design points from the original DOE matrix were considered real, whereas the points predicted from RSM were called virtual. The optimization results solution space is shown using 3D-bubble graph in Figure 14. It contains real and virtual design points, both feasible and unfeasible.

**Figure 15:**
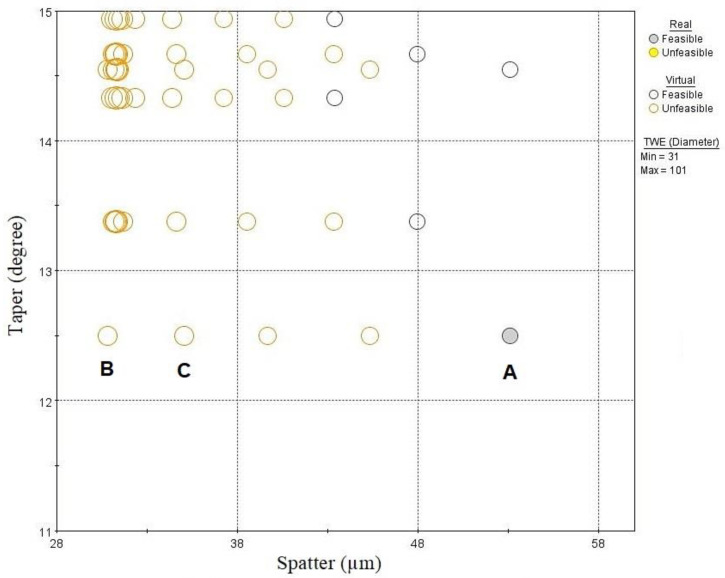
Optimal solution minimizing taper and spatter.

As the objective function is minimization of both taper and spatter, the optimal solution lies at the lower end of the solution space in Figure 14. The duplicate design points were deleted, and bubble chart is zoomed near the lower end in order to find the optimal design points as shown in Figure 15. It can be seen that least taper of 12.5 degree was achieved with one real feasible point A and few other infeasible points. However, within these design points spatter was found to vary significantly. Table 4 shows the selected optimum solutions; the optimal solutions consists of high frequency, medium to high speed, low layer-thickness and track displacement. It can be seen that points C is in the infeasible range because of the violation of only TWE constraint, but there was a significant reduction in spatter. Similarly point B violates both TWE and MRR constraint with little margin, but it could achieve best possible minimization of both taper and spatter. These solutions provide few choices for the decision-makers with some compromises in TWE and MRR.

## 4. Conclusions

In this work, laser-machining of microchannels in NiTi SMAs was investigated by varying five different process parameters: frequency, speed, layer thickness, track displacement and scan strategy. Optimal design IV-based experimental design methodology was used in order to investigate the effect of process parameters on the microchannel performance measures such as TWE, taper, spatter and MRR. A multi-objective optimization was performed in order to minimize the taper angle and spatter thickness using a combination of RSM and genetic algorithm. Based upon the analysis of results the following conclusions were drawn:

- The laser machined microchannels were associated with dimensional errors, tapered side walls and spatter. However, with suitable selection of process parameters these defects could be minimized;
- In general, higher values of speed and layer thickness, along with scan strategy S1 were found to produce microchannels with least dimensional error;
- Layer thickness was found to have a significant effect on the taper angle of the microchannel. Low layer-thickness resulted in microchannels with least taper angle;
- Spatter thickness was found to be significantly affected by scan speed, layer thickness and scan strategy. Low levels of speed and layer thickness along with scan strategy S1 was found to produced microchannels with least spatter;
- MRR was mostly influenced with scan speed, layer thickness and track displacement. Higher values of speed, layer thickness and track displacement result in higher MRR;
- Multi-objective optimization was successfully carried out using MOGA-II algorithm. Optimal solution was characterized by high frequency, moderate speed and low layer-thickness and track displacement;
- Optimization provides the decision-makers with the resourceful and efficient results. Optimal solutions could be selected from within these results depending upon the requirement;
- Optimal solution resulted in a minimum taper of 12.5° and a spatter of 30.8 µm. Additional experiments and optimization algorithms could be tested for further minimization of taper and spatter;
- Microhardness tests beneath the microchannel surface showed the absence of any detrimental effect of laser processing on the material properties. However, a detailed analysis of the effect of laser processing on the functional properties of the NiTi SMAs was essential for actual applications.

## Figures and Tables

**Table 1:** Factors and their respective levels.

Factor	1	2	3	4
Frequency	25 kHz	35 kHz	40 kHz	
Scan speed	200 mm/s	400 mm/s	600 mm/s	
Layer thickness	1 µm	2 µm	3 µm	
Track displacement	8 µm	10 µm	12 µm	
Scan strategy	S1	S2	S3	S4

**Table 2:** Design of experiment runs and experimental results.

Run	Frequency (kHz)	Speed (mm/sec)	Layer Thickness (µm)	Track Disp. (µm)	Scan Strategy	TWE (µm)	Taper (degree)	Spatter (µm)	MRR (µm^3^/s)
1	40	200	2	8	3	53.4	24.9	39.3	793,647.4
2	35	400	1	10	3	73.5	15.6	38.6	939,033.5
3	35	400	2	12	1	44.9	21.1	37.2	2,038,995
4	40	600	1	8	2	41.7	18.4	51.0	722,953
5	25	200	2	10	4	86.2	23.8	38.4	1,384,979
6	35	600	3	8	4	52.9	19.8	44.6	2,172,235
7	35	400	3	12	3	66.1	29.6	40.1	2,177,359
8	25	600	1	8	1	44.9	26.0	30.5	939,763.4
9	40	200	1	12	3	75.0	19.6	30.7	826,080.4
10	40	600	3	8	1	53.9	24.2	34.6	1,380,986
11	40	400	2	12	2	59.2	26.8	39.5	1,668,078
12	40	400	1	10	1	56.6	19.1	23.5	1,146,676
13	40	600	3	10	4	41.2	22.2	51.5	2,006,592
14	40	200	3	12	1	48.1	25.4	41.4	3,005,666
15	40	400	2	10	4	59.7	23.9	35.5	1,601,717
16	40	200	2	10	1	49.1	21.1	28.2	1,354,825
17	25	400	1	10	4	60.2	16.0	28.0	1,037,431
18	35	400	3	10	2	51.2	28.5	37.9	1,909,948
19	35	600	1	10	2	51.2	21.4	36.1	1,021,419
20	35	600	2	10	4	45.4	25.0	43.5	1,770,126
21	35	400	1	8	4	62.4	17.2	33.2	872,032.6
22	35	600	1	8	3	73.5	15.8	26.8	1,101,263
23	40	200	3	10	2	76.2	23.1	44.8	2,294,011
24	25	200	1	12	2	68.7	20.2	30.6	911,940.7
25	35	400	3	8	1	31.7	21.9	49.1	2,481,353
26	35	600	3	12	2	40.2	23.5	44.7	2,472,150
27	40	600	3	12	3	31.1	26.9	51.9	2,497,826
28	25	400	1	12	1	51.8	18.9	28.7	1,151,930
29	35	400	2	8	3	74.5	16.3	31.2	1,329,239
30	25	200	1	10	3	101.0	28.1	12.3	726,000.4
31	40	200	1	8	1	43.8	20.2	33.3	832,969.2
32	25	400	2	12	4	43.5	26.1	44.5	1,664,514
33	35	200	2	12	3	64.0	22.8	39.7	1,376,418
34	25	600	1	12	3	46.5	18.5	32.2	1,135,224
35	40	400	3	8	3	57.6	25.4	40.2	1,850,694
36	25	600	3	12	1	39.1	24.4	43.6	2,633,723
37	25	200	3	10	1	45.4	19.9	35.8	1,908,810
38	25	400	2	10	1	42.3	19.2	40.0	2,010,132
39	35	600	3	10	1	42.8	24.8	41.5	2,544,111
40	40	400	1	12	4	68.7	15.9	30.8	1,103,753
41	25	400	3	10	3	48.8	32.4	46.7	1,711,848
42	35	200	2	10	2	62.4	18.8	32.6	1,312,411
43	25	400	1	10	2	67.3	18.7	37.3	964,982.6
44	40	400	2	8	1	51.3	21.6	28.9	1,245,896
45	40	600	1	8	4	49.8	12.5	53.1	900,554.5
46	25	400	1	8	3	68.8	16.0	29.0	925,104.2
47	35	200	1	12	1	62.4	20.6	19.3	1,020,239
48	35	200	2	8	1	34.5	17.7	39.0	1,368,914
49	35	600	2	8	2	52.5	18.3	36.1	1,995,799
50	40	400	2	10	3	65.6	20.1	31.6	2,000,856
51	25	400	3	8	2	52.3	25.4	42.7	2,070,845
52	35	200	1	10	4	68.3	16.1	30.7	722,609.9
53	25	600	2	8	3	46.5	26.2	39.3	1,603,850
54	35	200	3	10	3	47.3	17.5	44.2	1,476,022
55	40	600	1	10	3	57.1	15.5	30.7	1,184,823
56	25	200	2	8	2	54.0	23.2	33.5	1,124,789
57	25	400	3	12	4	45.5	26.5	46.7	2,474,509
58	40	200	2	12	4	73.1	23.7	34.9	1,418,359
59	35	200	1	8	2	69.3	19.6	28.5	700,093.2
60	35	600	1	12	4	55.6	16.4	32.3	1,270,329
61	40	200	3	8	4	67.8	25.6	36.2	1,381,260
62	25	600	3	10	2	40.7	25.6	46.9	2,569,118
63	40	600	2	12	1	46.1	22.0	35.2	1,646,294
64	25	200	3	12	3	63.5	23.8	34.2	1,832,067
65	25	200	1	8	4	73.6	17.3	27.0	778,558.2

**Table 3:** Objective functions and constraints for the optimization.

Objective function	1. Minimize taper
	2. Minimize spatter
Constraints	1. TWE < 50 µm
	2. MRR > 900,000 µm^3^/s

**Table 4:** Optimum parameters minimizing taper angle and spatter of the microchannel.

ID	Frequency (kHz)	Speed (mm/sec)	Layer Thickness (µm)	Track Disp. (µm)	Scan Strategy	Taper (Degree)	Spatter (µm)	TWE (µm)	MRR (µm^3^/sec)	
A	40	600	1	8	4	12.5	53.1	49.8	900554.5	F
B	40	400	1	8	4	12.5	30.8	60.2	872032.6	NF
C	40	450	1	8	4	12.5	35.1	59.6	905973.3	NF
